# Paradoxical carbapenemase activity detected by modified carbapenemase inactivation (mCIM) method in *Citrobacter sedlakii*

**DOI:** 10.1128/jcm.00589-25

**Published:** 2025-08-25

**Authors:** D. Garrett Brown, Matthew Surette, Sreejana Ray, Kevin Guo, Reagan Chan, Liusheng Huang, Sanchita Das

**Affiliations:** 1 Department of Laboratory Medicine, National Institutes of Health (NIH) 2511, Bethesda, Maryland, USA; 2 Department of Clinical Pharmacy, University of California San Francisco 209683, San Francisco, California, USA; Maine Medical Center Department of Medicine, Portland, Maine, USA

**Keywords:** false-positive mCIM, antimicrobial resistance testing, *Citrobacter sedlakii*

## Abstract

Screening for carbapenemase-producing Enterobacterales is a necessity for hospitals serving high-risk patient populations. Detection methods often incorporate the modified carbapenemase inactivation method (mCIM) test to verify carbapenemase production in suspect isolates. Here, we demonstrate the case of a *Citrobacter sedlakii* isolate recovered from carbapenemase-surveillance culture that was strongly mCIM positive for carbapenemase production, but phenotypically nonresistant to carbapenems. The modified carbapenemase inactivation method with EDTA (eCIM) test, which suggests the presence of a metallo-β-lactamase, was positive as well. Liquid chromatography mass spectrometry additionally confirmed the loss of meropenem during the mCIM test. Whole-genome sequencing detected only the presence of the chromosomal class A serine β-lactamase SED-1 but did not identify any known carbapenemase or additional β-lactamases. Further characterization suggested that the large inoculum utilized in mCIM tests can give the impression of carbapenemase production in non-resistant isolates. Additionally, the large inoculum, combined with weak carbapenemase activity, can mimic the phenotype expected for metallo-β-lactamase in the eCIM test. These results suggest that utilization of the mCIM and eCIM tests without appropriate susceptibility testing can lead to erroneous detection of carbapenemase production.

**IMPORTANCE:**

Phenotypic tests including the mCIM are often used for detection of carbapenemase production in Enterobacterales. The presence of a carbapenemase gene is confirmed by PCR assays and, while rarely seen, a positive mCIM assay and negative PCR can alert the laboratory to the presence of rare carbapenemases (such as GES and IMI). We report a false-positive mCIM test, which we demonstrate is due to the heavy inoculum of the organism used in the assay. Whole-genome sequencing showed that a class A β-lactamase (*bla_SED-1_*) was present, and mass spectrometry demonstrated degradation of meropenem during the mCIM assay. The inoculum effect should be considered when interpreting mCIM and eCIM assays where a carbapenemase gene is not detected.

## INTRODUCTION

Carbapenemase-producing Enterobacterales (CPEs) are an ever-evolving threat to human health. Estimates suggest that these organisms may be directly attributable to over 200,000 deaths annually (1). In the hospital setting, CPE can transmit between patients, causing outbreaks of life-threatening disease (2–5). These organisms may be resistant to all β-lactam antibiotics and, due to the localization on plasmids with multiple resistance determinants, may also be resistant to alternative classes of antibiotics. Therefore, in hospitals serving high-risk patient populations, preventative screening for these organisms (2), as part of infection control programs, can reduce hospital-associated mortality and morbidity (6). Though antimicrobial susceptibility testing (AST) can identify phenotypic resistance, definitive identification of CPEs necessitates molecular, enzymatic, immunoassay, or modified carbapenemase inactivation method (mCIM) testing. Molecular and immunoassay testing is often limited to a predetermined set of target genes, while enzymatic assays may be expensive and difficult to interpret. Therefore, the mCIM is often employed as a broad, easy-to-use test for carbapenemase production. In this test, a loopful of the test isolate is incubated in liquid media with a meropenem disk. The disk is then placed on a lawn of carbapenem-susceptible *Escherichia coli*. If the meropenem is enzymatically degraded, the disk will not inhibit the growth of the indicator *E. coli*. Alternatively, if the meropenem is not degraded, a zone of inhibition of the *E. coli* will be observed around the disk. Discrimination between metallo-β-lactamase and serine β-lactamase is also possible, using the related modified carbapenemase inactivation method with EDTA (eCIM) method. This method incorporates the addition of EDTA to the initial incubation step, chelating metal ions and specifically inhibiting the function of metallo-β-lactamases. Isolates positive by eCIM (metallo-β-lactamase producers) are therefore prevented from inactivating meropenem, and the disk is able to inhibit the growth of the indicator *E. coli*.

As the name suggests, the mCIM is a modification of the originally described CIM test. The original CIM test involves the inoculation of a larger volume of cells into water with a meropenem disk (7). The water does not support the growth of the organism and, thus, assays for existing carbapenemase (and necessitates a larger inoculum and shorter incubation duration). The CLSI-endorsed mCIM test was developed for clinical application and illustrated an increased sensitivity, with similar specificity in Enterobacterales (8).

A member of the Enterobacteriaceae family, *Citrobacter sedlakii*, inhabits the mammalian GI tract and can be recovered when screening for carbapenem-resistant organisms. *C. sedlakii* can carry plasmids encoding resistance to carbapenems and other classes of antibiotics (9–11). A plasmid found in *C. sedlakii* carrying NDM was also identified in other Enterobacteriaceae species in the same sample, suggesting the possibility of horizontal transfer between this organism and other definitive pathogens (9). Furthermore, plasmids in *C. sedlakii* can be transferred to *E. coli*, as demonstrated by transfer of colistin resistance (10). *C. sedlakii* itself has also been associated with invasive infections (12–14).

Due to our high-risk patient population, we perform comprehensive screening for CPEs. These steps are designed to limit nosocomial transmission of antibiotic-resistant organisms, rather than strictly for patient care. Here we describe a *C. sedlakii* isolate (*C. sedlakii* NIH2339) identified in our hospital CPE screening program that appeared carbapenem resistant via growth on selective, chromogenic agar. In the mCIM test, it displayed strong meropenem degradation. The eCIM test was performed and suggested that the carbapenemase was a metallo-β-lactamase. However, via broth microdilution, the isolate remained nonresistant to all carbapenems tested. Further testing by whole-genome sequencing (WGS) did not identify a carbapenemase but confirmed the chromosomally encoded *bla_SED-1_* found in all *C. sedlakii* isolates. We further demonstrate a strong inoculum effect associated with this isolate, likely due to weak carbapenemase activity of SED-1, that can lead to false-positive eCIM and mCIM tests.

## MATERIALS AND METHODS

### Bacterial isolates

*C. sedlakii* NIH2339 was isolated from a patient undergoing routine carbapenemase susceptibility screening at the NIH Clinical Center. *K. pneumoniae* ATCC 1705 (KPC producing) and *K. pneumoniae* ATCC 1706 (meropenem susceptible) were utilized as controls in the mCIM tests (and its derivatives). *E. coli* ATCC 25922 was used as a susceptible strain for plating meropenem disks in the mCIM. NDM-producing *E. coli*, NDM-producing *Citrobacter amalonaticus* NIH1136, and carbapenem-susceptible *C. amalonaticus* NIH0944 were molecularly characterized clinical isolates.

### Carbapenemase screening

Routine carbapenemase screening is performed at the NIH Clinical Center to prevent and control outbreaks. Perirectal swabs are delivered to the laboratory, and swabs are vortexed in 1 mL saline. Approximately 100 µL inoculated saline is streaked for isolation on HardyCHROM CRE plates (Hardy Diagnostics). Any growth on these plates after 24 h of incubation at 35°C, ambient air, is characterized via matrix-assisted laser desorption/ionization time-of-flight (MALDI-TOF), broth microdilution antibiotic susceptibility testing, mCIM testing, and XPert Carba-R (Cepheid) testing to rule out carbapenemase-producing organisms. Workup of non-pink/blue colonies does deviate from the package insert for the HardyCHROM CRE plates.

### MALDI-TOF

Species identification was performed using the MALDI BioTyper SMART MS (Bruker). Growth on solid plates was spotted onto a BigAnchor MicroSCOUT plate, along with quality control (QC) organisms and negative controls. Seventy percent formic acid was overlayed on spots, followed by Bruker Matric HCCA. The spot was air-dried before running MALDI following the manufacturer’s instructions. Identification was confirmed with a score of ≥2 after comparison to a custom MALDI database.

### Antimicrobial susceptibilities

Broth microdilution (BMD) and disk diffusion susceptibility testing were performed and interpreted according to the procedures described in the CLSI M07 (12th edition), CLSI M02 (14th edition), respectively, and the CLSI M100 (32nd edition). BMD susceptibility testing was performed with GN7F and ESB1F TREK Sensititre panels (Thermo Fisher) and the ARIS-HiQ system according to the manufacturer’s instructions. Colonies were suspended in water and adjusted to a 0.5 McFarland standard. Thirty microliters of the standard was transferred to Mueller-Hinton broth, and 50 µL was added to each well of each Sensititre plate. The plates were incubated for 18 h and read automatically and manually. Additionally, the meropenem MIC was independently determined by the ETEST (BioMerieux). For disk diffusion testing, pure colonies were adjusted to a 0.5 McFarland standard in saline. A sterile cotton-tipped applicator was dipped in the suspension and inoculated onto a Mueller-Hinton agar plate. After the streak was dried, antibiotic-impregnated disks were laid on the plate.

### Carbapenemase PCR

The Xpert Carba-R (Cepheid) test was used to perform molecular testing for isolates that could be carbapenemase producers due to growth on HardyCHROM CRE plates. From pure colonies, a 0.5 McFarland preparation was generated, and 10 µL was mixed with the provided sample reagent, loaded into the Cepheid cartridge, and run via the manufacturer’s instructions.

### Modified carbapenemase inactivation method

mCIM testing was performed by inoculating 2 mL of tryptic soy broth (TSB) with 1 µL loop of organism, along with a 10 µg disk of meropenem, and incubating for 4 h at 35°C. After incubation, a 0.5 McFarland standard of *E. coli* ATCC 25922 was plated on Mueller-Hinton agar, and the meropenem disk was added on top of the plate. The Mueller-Hinton agar was then incubated at 35°C in ambient air overnight. The next day, the zone sizes were measured to determine carbapenemase activity. *Klebsiella pneumoniae* ATCC 1705 and 1706 were tested in parallel with each isolate to serve as positive and negative controls, respectively. To perform the eCIM test, the mCIM protocol was performed with the addition of EDTA (at a final concentration of 5 mM) to bacterial culture with the meropenem disk, as described in CLSI M100 (15). For eCIM testing specifically, *K. pneumoniae* ATCC 2146 was included as a positive control.

### Carbapenemase inactivation method

As a growth-independent measure of carbapenemase activity, we performed the original carbapenemase inactivation method (CIM) (7). More specifically, 400 µL of sterile deionized H_2_O was inoculated with 10 µL loopfuls of bacteria and a 10 µg meropenem disk with or without EDTA (50 mM final concentration). After 2 h, the disk was removed and plated on a lawn of susceptible *E. coli*, as performed in the mCIM test. The plate was read after overnight incubation.

### Liquid chromatography-tandem mass spectrometry

To quantify meropenem in the mCIM medium, at the end of the mCIM incubation period (after 4 h of growth), the culture was centrifuged at 5,000 × *g* for 10 minutes, and the supernatant was filtered through a 0.2 µm filter to remove whole bacterial cells. The supernatant was frozen at −20°C until further processing. Quantification of meropenem was carried out at the Drug Research Unit at the University of California San Francisco based on a published method with modification (16). Briefly, the calibrator and QC samples were spiked in water from the same stock solution used for the incubation experiment. Spiked samples, along with the *in vitro* samples, were diluted by 40-fold with water and injected onto a liquid chromatography-tandem mass spectrometry (LC-MS/MS) system.

### DNA isolation

Genomic DNA was isolated from *C. sedlakii* NIH2339 for both short-read WGS and long-read WGS. For the short read WGS, the bacterial isolate was subcultured and grown in 2 mL TSB overnight (14–16 h), and total nucleic acids were extracted using the MasterPure DNA & RNA Purification Kit (Lucigen) following the method as described (17). For long-read WGS, genomic DNA was isolated using the Promega Wizard HMW DNA isolation kit following the manufacturer’s protocol.

### DNA library preparation and whole-genome sequencing

The Illumina (short-read) sequencing library was prepared from 25 ng of DNA using the Nextera XT kit with slight modifications, as described previously (17). The purified library was quantified with Qubit 2.0, normalized to 4 nM and stored at −80°C until sequencing. QC was performed on the library using a 2100 Bioanalyzer (Agilent Genomics), and the resulting library was sequenced on Illumina NextSeq 500/550 with 2 × 150 bp paired-end reads. For the long-read WGS, a library was prepared using 200 ng of isolated DNA following the Nanopore Rapid barcoding kit protocol. QC of the generated library was done using Qubit 2.0 and 4150 Tape Station System (Agilent Genomics). The long-read WGS was performed on a Nanopore Gridiron sequencer using a Flongle flow cell (v.R10, FLO-FLG114).

### Hybrid genome assembly, species identification, and antimicrobial resistance gene identification

The raw sequencing data after the short-read WGS were basecalled using NextSeq 500/550 Default bcl2fastq software. Quality assessment of the data was performed on the NIH HPC Biowulf cluster (https://hpc.nih.gov) using FastQC (v.0.11.9) and multiQC (v.1.9) (18). Adapter sequences were removed with Trimmomatic (v.0.40) (19). Genome assembly was conducted *de novo* using SPAdes (v.3.15.5) (20), and the assembled genomes underwent thorough quality evaluation using FastQC, multiQC, and the Bacterial and Viral Bioinformatics Resource Center (BV-BRC) (https://www.bv-brc.org/) (21). For the long-read WGS, raw data QC, adapter trimming, and genome assembly were done using EPI2ME software by Oxford Nanopore Technologies.

To obtain a complete and accurate whole-genome sequence, both short-read and long-read assembled genomes were used to generate a hybrid assembled genome using Unicycler (v.0.4.8), on the NIH HPC platform (22). Unicycler integrates SPAdes.py (v.3.15.5) Miniasm and Minimap to generate the hybrid assembly from both long-read and short-read sequences and finally polishes the assembled sequence using Recon (v.1.4.3) and Pilon (v.1.23). Comparison of the *bla_SED-1_* region genomic context from publicly available *C. sedlakii* genomes (GCF_902377535.1, GCF_965181595.1, GCF_000759835.1, and GCF_035024825.1) was performed using clinker (v.0.0.31) (23). Visualization of De Bruijn assembly graphs was performed using Bandage (v.0.9.0) (24). Trimmed Illumina reads were aligned to our proposed *bla_SED-1_* region from *C. sedlakii* NIH2339 using bwa-mem2 (v.2.2.1) (25). Sorted BAM files were constructed using SAMtools (v.1.21) (26) and visualized in Integrated Genome Viewer (27). We reconstructed our proposed sequence for the *bla_SED-1_* region in *C. sedlakii* NIH2339 by concatenating two consecutive copies of contig 2 with the flanking chromosomal regions from contig 1 to either side.

To identify species, assembled contigs from both sequencing methods were indexed and analyzed using average nucleotide identity (ANI) and a tetra correlation search via the JSpeciesWS tool (28). This was further confirmed by species validation through PubMLST (29), based on ribosomal protein subunit genes. Detection of antimicrobial resistance genes was performed using the Resistance Gene Identifier (RGI) (v.6.0.3) pipeline, in conjunction with the Comprehensive Antibiotic Research Database (v.3.2.9) (30, 31).

## RESULTS

### *C. sedlakii* NIH2339 demonstrates questionable carbapenemase activity

**Fig 1:**
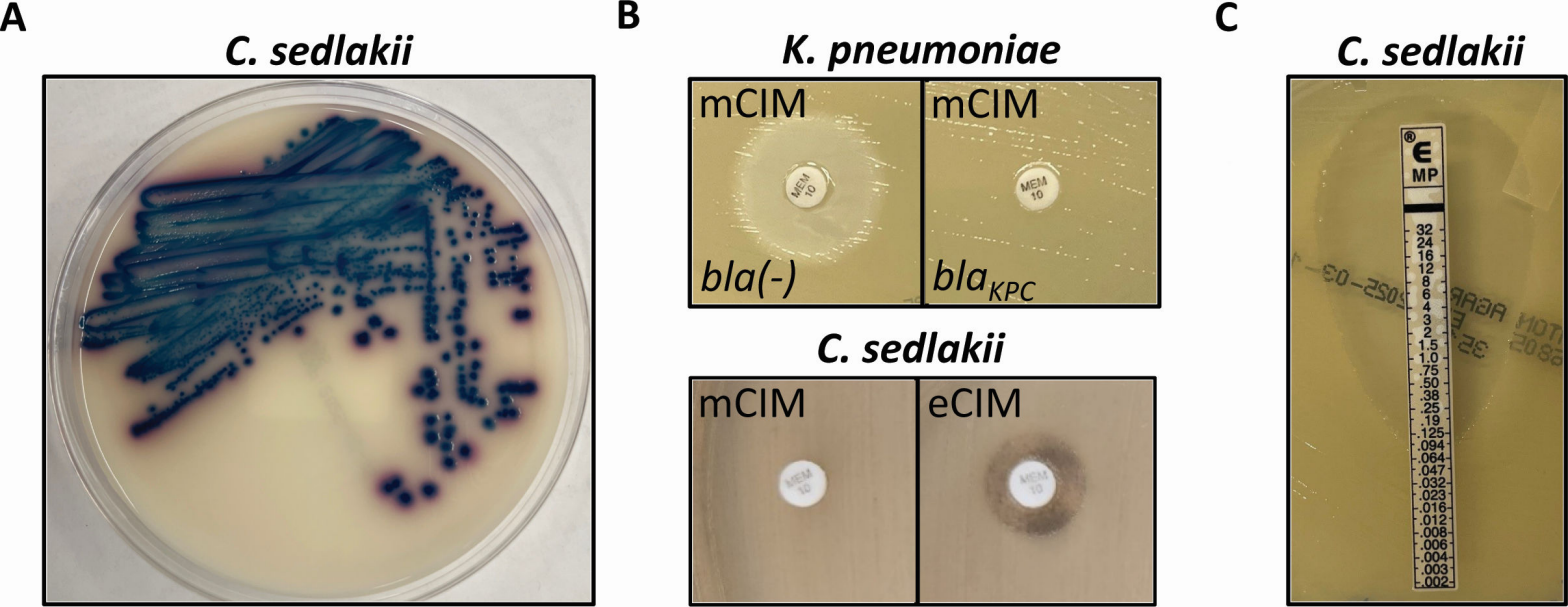
Screening suggests Ambler class B carbapenemase production in *C. sedlakii* NIH2339. (**A**) *C. sedlakii* NIH2339 growth on chromogenic CRE selective plate. (**B**) Growth of indicator *E. coli* with meropenem disks from the mCIM/eCIM tests. (Top left) A disk incubated with non-carbapenemase-producing *K. pneumoniae* strain 1706. (Top right) A disk incubated with KPC-producing *K. pneumoniae* strain 1705. (Bottom) A disk from mCIM (bottom left) and eCIM (bottom right) incubated with *C. sedlakii* NIH2339. (**C**) Meropenem E-test strip on *C. sedlakii* NIH2339.

Upon CPE surveillance screening, a patient admitted to the hospital was found to be colonized with an organism growing as blue colonies with a pink halo on HardyCHROM agar (Fig. 1A). MALDI-TOF identified this isolate as *C. sedlakii* (referred to as *C. sedlakii* NIH2339). Following our screening protocol, the Xpert Carba-R PCR (Cepheid) assay was performed on *C. sedlakii* NIH2339 and did not identify any of the carbapenemase targets (OXA-48, VIM, IMP, KPC, and NDM). An mCIM test was subsequently performed, demonstrating no zone of inhibition (Fig. 1B), which suggests the degradation of meropenem by this isolate. To understand if the carbapenemase activity was mediated by a metallo-β-lactamase, we performed the eCIM test. The eCIM is recommended by CLSI to differentiate serine and metallo-β-lactamases if mCIM results are positive (15). When EDTA was added to the medium, we observed a dramatic increase in zone diameter (6–14 mm) (Fig. 1B), ostensibly suggesting that the enzyme was dependent on metal cations and potentially a metallo-β-lactamase.

As our screening methods are designed to most quickly determine the presence of carbapenemases to inform infection control, we performed Carba-R and mCIM testing before ASTs. If either method produced a positive result, we then phenotypically confirmed results with AST. In stark contrast to the results of the mCIM, broth microdilution susceptibility testing demonstrated the isolate was susceptible to meropenem, doripenem, and imipenem, and intermediate to ertapenem (Table 1). For select carbapenems and cephalosporins, Kirby-Bauer disk diffusion was additionally performed (Table S1). We confirmed phenotypic susceptibility to meropenem using a second method, an E-test, which demonstrated a MIC of 0.094 µg/mL (Fig. 1C). This isolate was, however, resistant to cephalosporins but susceptible to ceftazidime-avibactam, suggesting the presence of an Ambler class A cephalosporinase.

**TABLE 1:** Broth microdilution susceptibility testing results of *C. sedlakii* NIH2339 to β-lactam antimicrobial agents

Antibiotics	BMD^*c*^ MIC	Interpretation^*d*^
Penicillins		
Ampicillin	>16	R
Ampicillin/sulbactam	>16/8	R
Piperacillin/tazobactam	>64/4	R
Monobactams		
Aztreonam	>16	R
Cephalosporins		
Cefazolin	>16	R
Ceftazidime	>16	R
Ceftazidime/avibactam	≤2	S
Ceftriaxone^*a*^	>128	R
Ceftolozane/tazobactam	>16	R
Cefepime	>16	R
Cefotaxime^*a*^	>64	R
Cefotaxime/clavulanic acid^*a*^	>64	NI
Cefoxitin^*a*^	64	R
Cefpodoxime^*a*^	>32	R
Ceftazidime/clavulanic acid^*a*^	64	NI
Cephalothin^*a*^	>16	NI
Carbapenems		
Doripenem	≤0.5	S
Ertapenem	1	I
Imipenem^*a*^	≤0.5	S
Meropenem^*b*^	≤0.5	S

^*a*^ BMD results from ESB1F plate.

^*b*^ Meropenem MIC of 0.094 via gradient strip testing.

^*c*^ BMD, broth microdilution.

^*d*^ R, resistant; S, susceptible; I, intermediate; NI, no interpretation. Interpretations are based on CLSI M100.

### LC-MS/MS confirms meropenem degradation by *C. sedlakii* NIH2339

We performed further testing to mediate the discrepancy of an organism that is phenotypically susceptible to carbapenems but potentially degrading meropenem in the mCIM test. Specifically, we performed LC-MS/MS analysis of meropenem in the supernatant from the broth incubation step of the mCIM to assess the concentration of meropenem remaining in the broth. We found that the supernatant from *C. sedlakii* NIH2339 culture contained a 92% reduction of meropenem, compared to a control containing a meropenem disk and no bacteria (Table 2). When the supernatant from the KPC-containing *K. pneumoniae* ATCC 1705 was analyzed, the medium contained no detectable meropenem. Incubation with the non-carbapenem-degrading *K. pneumoniae* 1706 illustrated only a 23% reduction compared to the disk alone. As with routine mCIM testing, the meropenem disks were placed upon a lawn of *E. coli*. Results were similar to our original mCIM tests, with no zone of inhibition around the disk incubated with *C. sedlakii* NIH2339 (not shown). Despite its relative phenotypic susceptibility, the marked depletion of meropenem from culture media during the mCIM test as measured by both bioactivity and LC-MS/MS is strong evidence that *C. sedlakii* NIH2339 produces a carbapenemase.

**TABLE 2:** LC-MS/MS to quantify meropenem in bacterial supernatant

Sample tested^*a*^	Mean MEM^*b*^ concentration (ng/mL)	SD	CV%
TSB (media)	0	NA^*c*^	NA
*C. sedlakii* NIH2339	0	NA	NA
MEM disk	4,443	398	9.0
*K. pneumoniae* ATCC 1706 + MEM disk	3,403	430	12.6
*K. pneumoniae* ATCC 1705 + MEM disk	0	NA	NA
*C. sedlakii* NIH2339 + MEM disk	374	40	10.7

^*a*^ All tests were performed in triplicate.

^*b*^ CV%, coefficient of variation; MEM, meropenem; SD, standard deviation.

^*c*^ NA, not applicable.

### No carbapenemase encoding genes identified by whole-genome sequencing

*C. sedlakii* are known to harbor a chromosomally encoded class A β-lactamase, *bla_SED-1_*, which is not known to possess carbapenemase activity (32). Therefore, to identify if any additional β-lactamase genes were responsible for the observed activity, we performed whole-genome sequencing on this isolate. The average DNA library size generated for short-read WGS was 550 bp, and after the sequencing, Illumina NextSeq 550 generated 4,604,091 raw reads. During the initial QC using FastQC and MultiQC, raw reads were processed to trim the adapters and poor reads. QC was done also after the genome assembly using both FastQC and BV-BRC platform. The assembled genome contained 35 contigs with an average sequencing depth of 166×. The average DNA library size generated for long-read sequencing using Nanopore Rapid barcoding kit was 10 kb. WGS by Nanopore GridION generated 3,003 reads with a mean read length of 10,090.6 bases. The hybrid assembly of the genome combining both short-read and long-read WGS reduced the total number of contigs to just 3. Contigs 1 and 2 are linear contigs of 4.6 Mb and 17.7 kb, respectively. Contig 3 is a 4.4 kb circular molecule greater than 99.9% identical to a plasmid found in *K. pneumoniae* (pKPNB_13889.4, GenBank: CP154157.1) and other Enterobacteriaceae. The ANI-based alignment free genome identity analysis using JSpeciesWS online tool confirmed the identification as *Citrobacter sedlakii* after the genome assembly. Lastly, we compared our genome to a reference *C. sedlakii* assembly (GCA_902377535.1) using BV-BRC, which showed our genome was 99.9% complete with 0.1% contamination and therefore suitable for further analysis.

Next, we utilized the Resistance Gene Identifier and the Comprehensive Antibiotic Resistance database to identify resistance determinants. Via this method, we identified the presence of *bla*_*SED-1*_, the gene that encodes the SED-1 chromosomal β-lactamase, but no additional β-lactamases that could contribute to meropenem degradation. No resistance genes were identified on the small plasmid (contig 3). Intriguingly, RGI identified *bla_SED-1_* on the linear 17.7 kb contig 2, and no β-lactamase resistance genes were identified in contig 1. We noted that contig 2 is sequenced at 8.56-fold higher depth than contig 1, suggesting that multiple copies of this region are present in the genome. More detailed analysis of the genomic context and assembly shows that successive duplications have taken place at this chromosomal locus (Fig. S1), potentially leading to higher levels of *bla_SED-1_* expression. Meropenem degradation in this isolate could therefore arise through weak carbapenemase activity of SED-1, which would be a novel finding, detected in our isolate due to potential overexpression. Given that *C. sedlakii* NIH2339 is also eCIM positive, we reasoned that meropenem degradation could also be mediated by the presence of a novel or cryptic metallo-β-lactamase.

### Additional testing confirms the presence of a non-metallo-β-lactamase carbapenemase

**Fig 2:**
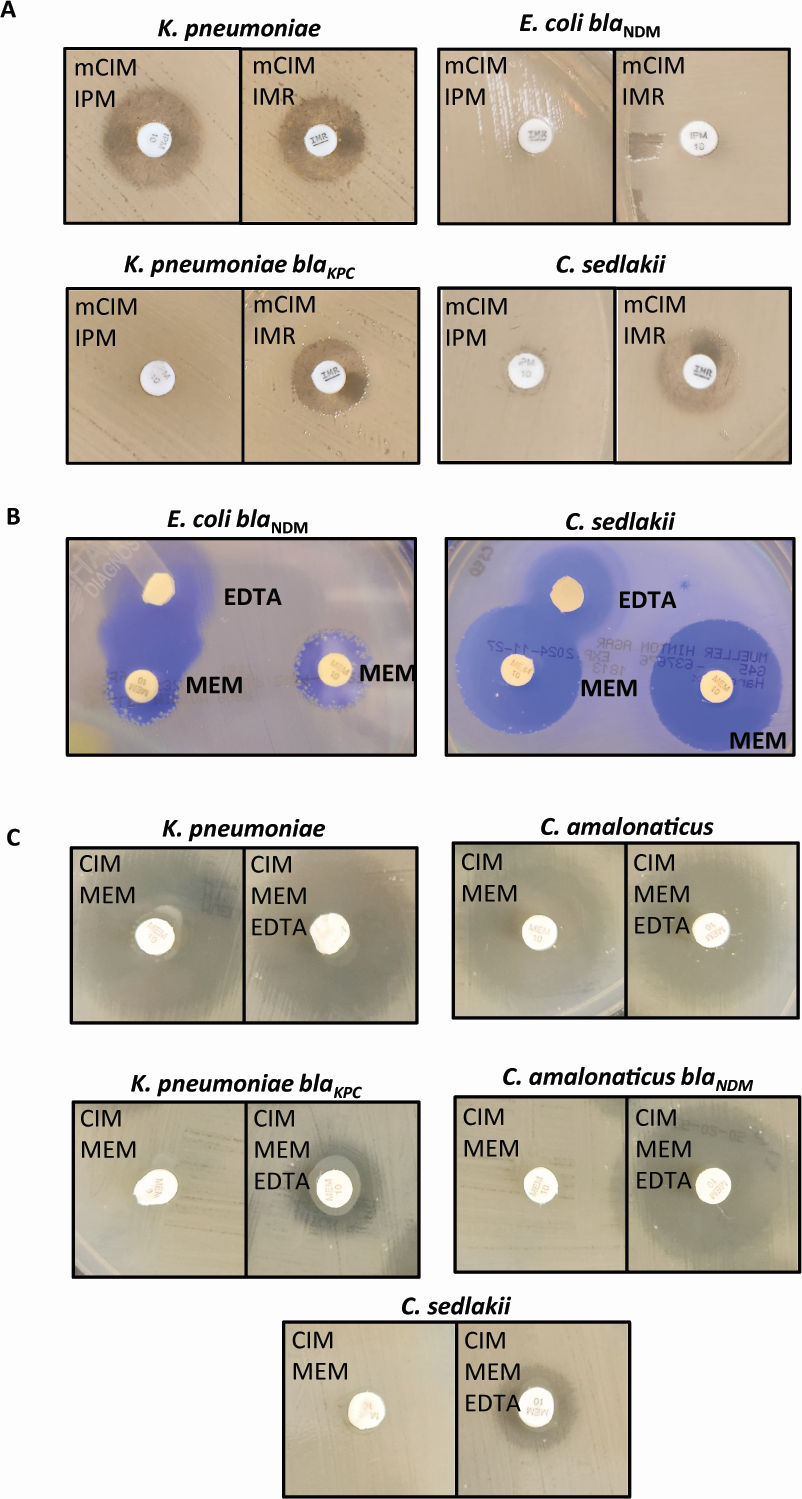
*C. sedlakii* NIH2339 possesses serine carbapenemase activity. (**A**) mCIM test with imipenem (left) or imipenem-relebactam (right) disks, using *K. pneumoniae* ATCC 1706, NDM-*E. coli*, KPC-producing *K. pneumoniae* ATCC 1705 or *C. sedlakii* NIH2339. (**B**) Test for interaction between a disk containing EDTA and meropenem with NDM-*E. coli* (left) or *C. sedlakii* NIH2339 (right). (**C**) CIM test with EDTA (right panels) or without EDTA (left panels), testing *K. pneumoniae* ATCC 1706, non-carbapenemase-producing *C. amalonaticus*, KPC-producing *K. pneumoniae* ATCC 1705, NDM-producing *C. amalonaticus*, and *C. sedlakii* NIH2339. IPM, imipenem; MEM, meropenem.

We hypothesized that if SED-1 was responsible for carbapenemase activity in *C. sedlakii* NIH2339, then a broad-spectrum serine β-lactamase inhibitor should block carbapenem degradation in the mCIM test. Conversely, if a cryptic metallo-β-lactamase was present, its activity should be unaffected. We therefore performed an additional mCIM test, instead utilizing disks of imipenem and imipenem relebactam. As we observed with meropenem, *C. sedlakii* NIH2339 displayed complete inactivation of imipenem during the mCIM test, but intriguingly, the zone diameter increased to 15 mm when relebactam was included (Fig. 2A). Simultaneous testing of an NDM-producing *E. coli* shows no increase in zone diameter when relebactam is added. As relebactam inhibits class A, C, and D β-lactamases (33, 34), our observation that relebactam can block carbapenemase activity in this isolate suggests that there is no metallo-β-lactamase and that the chromosomally encoded SED-1 most likely possesses weak carbapenemase activity.

Because *C. sedlakii* NIH2339 is positive by the eCIM test, we sought to rule out the production of a metallo-β-lactamase through additional experiments. First, we tested for the potentiation of meropenem by subinhibitory concentrations of EDTA, which should occur in the context of metallo-β-lactamase production. This phenotype is readily observable on solid media when meropenem and EDTA-containing disks are placed nearby on a lawn of bacteria. For instance, the zone of inhibition is markedly larger in an NDM producer where the diffusing EDTA and meropenem meet (Fig. 2B). Using the same methodology, *C. sedlakii* NIH2339 demonstrated no such synergy.

Additionally, we performed a CIM test (using water instead of broth for incubation), with or without the addition of EDTA to the inoculum (Fig. 2C). In the CIM test, the isolate in question is added to deionized H_2_O with a meropenem disk. As bacteria are not actively growing, the degradation of meropenem is solely dependent on the activity of pre-formed enzyme. In the CIM assay, we observed the same robust carbapenemase activity in *C. sedlakii* NIH2339 as in the mCIM. When EDTA was added to the reaction, we observed an increase in the zone of inhibition (6–12 mm). This is similar to what was observed when a KPC-producing *K. pneumoniae* was tested (6–12 mm) and is likely attributable to the cytotoxic effects of the higher concentration (50 mM) EDTA on the indicator *E. coli* rather than inhibition of a metallo-β-lactamase. In contrast, we tested an NDM metallo-β-lactamase-producing *C. amalonaticus* isolate, which showed a drastic increase in zone size following EDTA addition (6–24 mm). Concurrently, carbapenem-susceptible *K. pneumoniae* and *C. amalonaticus* had large zone diameters with (24 and 27 mm) or without EDTA (24 and 25 mm). Taken together, these results suggest there is no production of a metallo-β-lactamase by *C. sedlakii* NIH2339.

### The inoculum effect causes discordance between phenotypic AST and mCIM results

**Fig 3:**
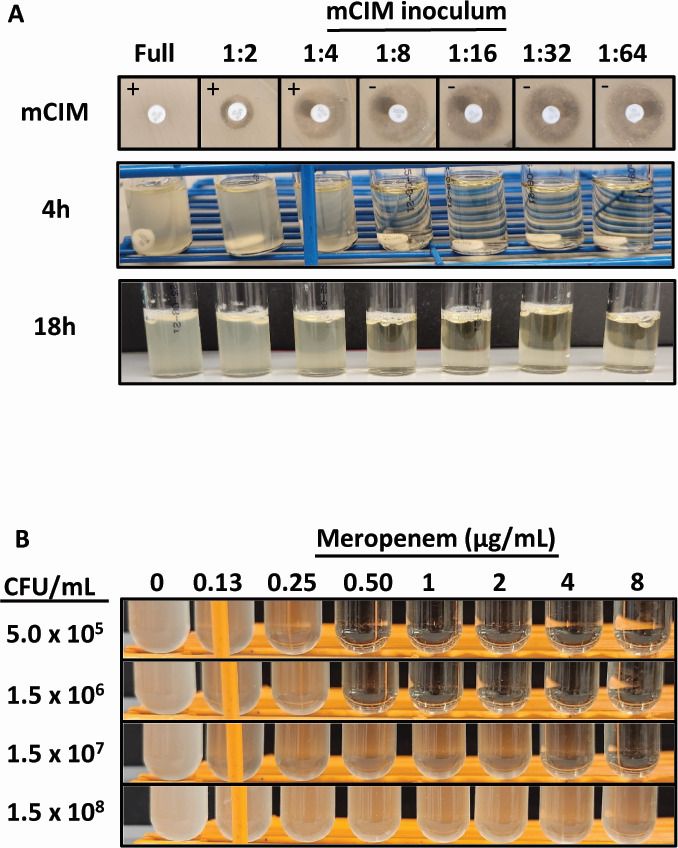
Carbapenemase-positive results are due to high inoculum. (**A**) mCIM results with different starting concentrations of *C. sedlakii* NIH2339. (Top row) A disk on susceptible *E. coli* (categorical interpretation noted in panels). (Middle row) Growth in tubes at 4 h (time point in which disk is removed for testing). (Bottom row) Growth if disk is kept in tubes for 18 h. (**B**) Growth of *C. sedlakii* in meropenem-containing media at different inocula and meropenem concentrations.

We hypothesized that the heavy inoculum used in the mCIM test, as compared to broth microdilution AST, could create the correct conditions for the SED-1 enzyme to degrade meropenem. With a higher inoculum, enough enzyme may exist in the culture medium or may be produced during the 4 h incubation to substantially deplete meropenem. To test this, we repeated the mCIM test inoculated with serial twofold dilutions of *C. sedlakii*. At the typical 4 h time point, the disks were removed and placed on a lawn of susceptible *E. coli*. We observed subsequent increases in zone diameter up to the 1:8 dilution. Beyond this dilution, the zone diameter did not increase further (Fig. 3A). At the 4 h time point, we also observed no growth at the 1:8 dilution or lower. To simulate a broth dilution assay, the tubes were held for 18 h, but even at that time point, we did not observe any bacterial growth beyond the 1:8 dilution of the original suspension used for mCIM. We then utilized broth dilution to measure the MIC of the *C. sedlakii* to meropenem with variable inoculum sizes, ranging from 1.0 × 10^8^ CFU/mL (similar to the concentration of cells used in the mCIM testing; we measured 2.8 × 10^8^ CFU/mL of *C. sedlakii* in the mCIM inoculum) to 5.0 × 10^5^ CFU/mL (the approximate concentration of cells used for broth microdilution testing). As the inoculum decreased, the MIC decreased as well, ranging from >8.0 to 0.25 µg/mL from the highest to lowest (Fig. 3B). These data suggest that the large inoculum in the mCIM is permissive to allow *C. sedlakii* NIH2339 growth and subsequent meropenem degradation.

## DISCUSSION

Hospitals, epidemiologists, and public health laboratories often depend on the mCIM test to prove phenotypic activity of carbapenemase activity. This is especially important in cases where carbapenemases present are not included as targets in commonly used PCR assays (such as GES and SPM). Several iterations of the mCIM assay are now published and have been extensively used due to the near-perfect sensitivity and specificity compared to molecular detection of carbapenemase genes (35). Since the adoption of this assay by CLSI in 2017, there have been few reports of false-positive results with the phenotypic carbapenemase assays, which have been attributed to the presence of chromosomal AmpC β-lactamase, mostly in *Enterobacter cloacae* complex (8). Importantly, however, SED-1 is class A β-lactamase, unlike AmpC, a class C β-lactamase.

Here, we illustrate that heavy inoculum used in the mCIM test can falsely suggest strong carbapenemase activity, when an organism is phenotypically susceptible to carbapenems and does not genotypically possess any known carbapenemase genes. Our experience demonstrates the necessity of phenotypic susceptibility testing to confirm the accuracy of mCIM results. We additionally found that the eCIM test can falsely suggest the presence of a metallo-β-lactamase. While the test is designed to utilize EDTA to chelate ions and inhibit class B carbapenemases, the addition of EDTA to the meropenem-containing medium might work additively to inhibit or kill cells, resulting in lack of carbapenem degradation and incorrect interpretation of resistance. False-positive eCIM tests are rare; in the original publication describing the eCIM test (36), the specificity was 100%.

Our data unexpectedly suggest that the SED-1 β-lactamase has a modest ability to degrade carbapenems. SED-1 is notable in its homology to the class A β-lactamases (like CTX-M) but is arranged with an upstream LysR repressor in an inverted orientation, like a class C (AmpC) β-lactamase (32, 37). The pattern of antimicrobial susceptibility in this isolate agrees well with the expected profile of an organism with a class A β-lactamase. It is tempting to speculate that in the correct genetic context, SED-1 may become hyperproduced in a similar manner to AmpC enzymes. In this isolate specifically, we uncovered evidence that a 17.7 kb region in the chromosome containing *bla_SED-1_* and *sedR* has been duplicated multiple times (Fig. S1), another potential route to high-level *bla_SED-1_* expression. The hyperproduction of a weak carbapenemase (SED-1) offers a satisfying rationale for the phenotypes we observed in this study. However, this level of mechanistic study was beyond the scope of the current work. Future studies should aim to characterize any carbapenemase activity of SED-1 biochemically and genetically. As other isolates of *C. sedlakii* have illustrated carbapenem susceptibility, it remains to be determined if all *C. sedlakii* isolates will show carbapenemase activity via the mCIM test or if our isolate has a gain of function (38). We identified polymorphisms in our SED-1 gene compared to other *C. sedlakii* genomes on NCBI, but no non-synonymous mutations were identified in it or its repressor, SedR. No quantitative evaluation of SED-1 expression was performed to determine if overexpression was occurring. Our study is limited in that we did not further analyze the β-lactamase to elucidate the inoculum effect on the carbapenem degradation. Further studies are needed to deductively investigate the activity of SED-1.

This study is the first (to our knowledge) to identify a false-positive eCIM due to a serine β-lactamase. We also illustrated the ability of the inoculum effect to produce false-positive results with two phenotypic carbapenemase assays, which can lead to significant downstream efforts in the clinical laboratory to identify the gene and to explain the genotypic/phenotypic discrepancy. When utilized for hospital infection control purposes. However, the mCIM still exists as an inexpensive, useful means of informing the laboratory on which isolates to investigate as carbapenemase producers. Due to its high sensitivity and specificity, the mCIM should still be considered as a pragmatic epidemiological tool.

CLSI AST guidelines for clinical purposes suggest that the mCIM should be performed on isolates that have first tested carbapenem non-susceptible. Given that workflow, positive mCIMs in carbapenemase-susceptible organisms would not occur. However, due to the higher cost and resource burden of broth microdilution ASTs, this workflow is likely not a feasible option in institutes that need to perform widespread screening for asymptomatically colonized patients.

This work has potential implications for patient outcomes in the case of a high bacterial burden such as in an abscess or a sequestered infection or in case a low dose of carbapenem was used inadvertently. The inoculum effect has been illustrated to occur with other β-lactam drugs, but carbapenems are generally observed to be less susceptible (39). Importantly, there are limited data available for the inoculum effect in *Citrobacter* spp. However, in another study, one *Citrobacter freundii* isolate (along with other Enterobacterales isolates) illustrated minimal increases in MIC for meropenem when the inoculum was increased from 10^5^ to 10^7^ CFU/mL while exhibiting large increases for cephalosporins and other β-lactams (40). Further work should determine if higher bacterial load in carbapenem-susceptible *Citrobacter* infection is associated with worse outcomes after carbapenem treatment.

Additionally, this work illustrates the utility of quantitative LC-MS/MS in the confirmation of carbapenemase activity. Previous work has demonstrated the utility of mass spectrometry in identifying carbapenemase production (41–43), but this is the first time, to our knowledge, that LC-MS/MS has been used to confirm the observed mCIM results. The benefit of the quantitative LC-MS/MS is illustrated in this case, where partial hydrolysis of a carbapenem is observed. While this is potentially difficult to implement in a clinical laboratory, it might have the ability to provide further information about new enzymes and their ability to degrade carbapenems.

### Conclusion

Inoculum effect was determined to be the cause of false-positive mCIM and eCIM tests in a *Citrobacter sedlakii* isolate producing a serine β-lactamase without substantial carbapenemase activity. We further show increased MIC to meropenem in this isolate related to a higher inoculum size, a feature that has observable *in vitro* implications and potential *in vivo* implications.

## SUPPLEMENTAL MATERIAL

Figure S1 and Table S1 jcm.00589-25-s0001.docx Genomic context of SED-1 in whole genome sequence and additional antimicrobial susceptibility results.

ASM does not own the copyrights to Supplemental Material that may be linked to, or accessed through, an article. The authors have granted ASM a non-exclusive, world-wide license to publish the Supplemental Material files. Please contact the corresponding author directly for reuse.
