# Supplementary material for: Paradoxical carbapenemase activity detected by modified carbapenemase inactivation (mCIM) method in Citrobacter sedlakii
Source: J Clin Microbiol. 2025 Aug 25;63(10):e00589-25. doi: 10.1128/jcm.00589-25 (PMC12506007; doi:10.1128/jcm.00589-25)
Supplement: Figure S1 and Table S1 — Genomic context of SED-1 in whole genome sequence and additional antimicrobial susceptibility results. [file jcm.00589-25-s0001.docx]

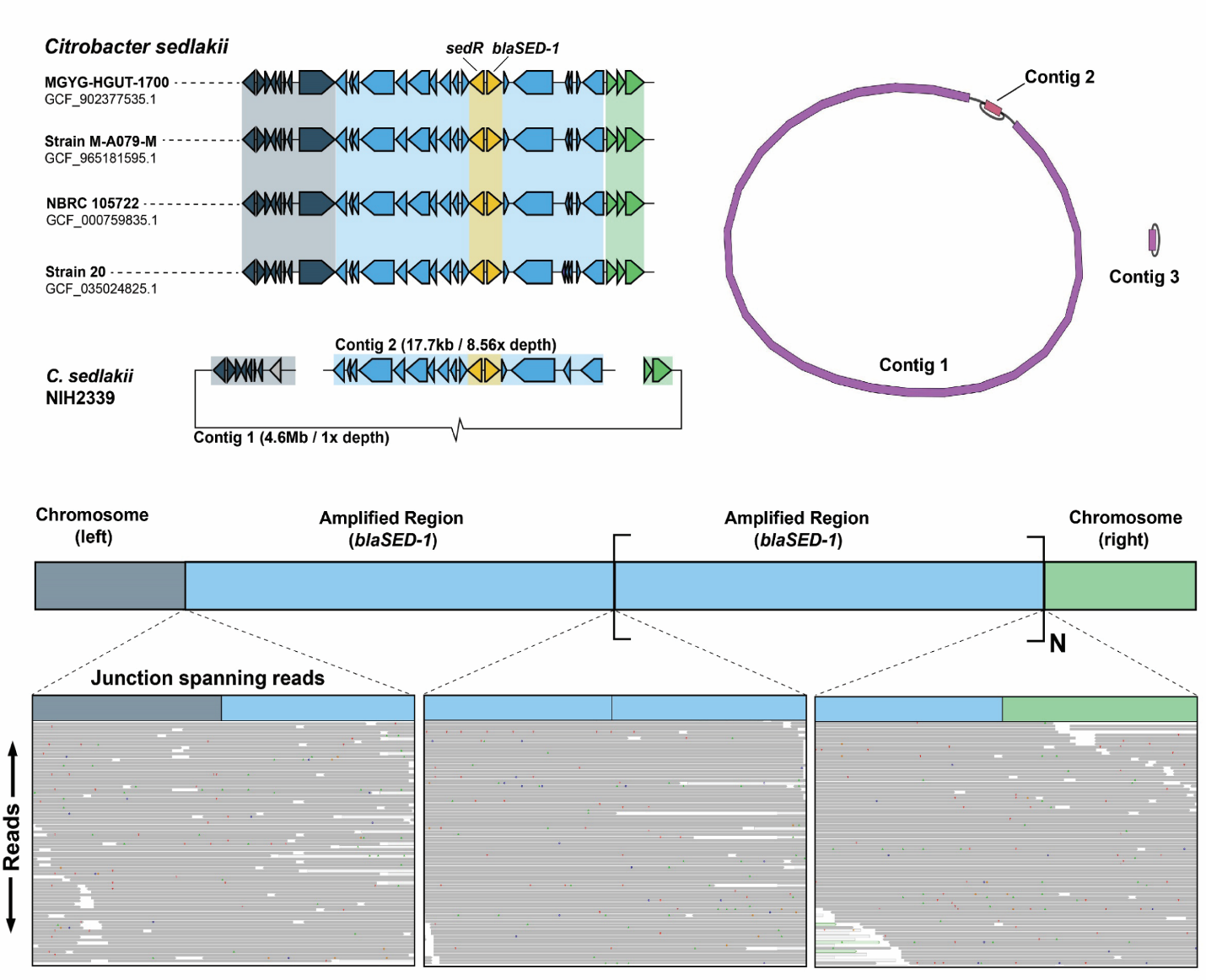


**Figure S1: Genomic context of *bla_SED-1_* in *C. sedlakii* NIH2339 shows evidence of amplification*.*** A) Comparison of the chromosomal *bla_SED-1_* region in various *C. sedlakii* whole genomes. In the *C. sedlakii* NIH2339 assembly this region is split across contig 1 which contains the bulk of the chromosome and contig 2 containing *bla_SED-1_.* Total sequencing depth for contig 2 is 8.56 times higher than the rest of the chromosome indicating there are multiple copies of this region in *C. sedlakii* NIH2339. B) De Brujin assembly graph depicting the connections contig 2 has with itself and the ends of contig 1.

C) Proposed architecture of the *bla_SED-1_* region in *C. sedlakii* NIH2339 featuring sequential duplications of a 17.7kb locus (contig 2). Illumina reads that span the junctions between the flanking chromosomal regions and the junction between successive amplified regions (contig 2) offer support for this architecture in *C. sedlakii* NIH2339.

**Table S1**. Disk diffusion susceptibility testing results of *C. sedlakii* NIH2339 to β-lactam antimicrobial agents.

| **Drug** | **Diameter (mm)** | **Interpetation** |
| --- | --- | --- |
| Ertapenem | 21 | I |
| Imipenem | 29 | S |
| Meropenem | 26 | S |
| Ceftazidime | 6 | R |
| Ceftazidime/Avibactam | 24 | S |
| Cefotaxime | 6 | R |
| Cefotaxime/Clavulanate | 6 | R |
